# Tissue Engineering: From Basic Sciences to Clinical Perspectives

**DOI:** 10.1155/2017/8659036

**Published:** 2017-01-23

**Authors:** Pornanong Aramwit, Antonella Motta, Subhas C. Kundu

**Affiliations:** ^1^Chulalongkorn University, Bangkok, Thailand; ^2^University of Trento, Trento, Italy; ^3^Indian Institute of Technology Kharagpur, Kharagpur, India; ^4^3B Research Group, University of Minho, Braga, Portugal

Tissue engineering and regenerative medicine are interrelated terms and go hand in hand whether we discuss about cells (of any kind especially stem and progenitor cells), biomaterials as matrices (2D films, 3D forms of scaffolds, nanofibers, hydrogels, nanoparticles, aerogel, microcapsules, mats, biogel for 3D printing, blends of naturals and/or synthetics, and others), and addition of bioactive molecules (delivery of growth hormones and drugs) for improvement and/or regeneration of tissues for biomedical applications (in relation to cartilage, shin, bone, blood vessels, nerve conduits, cardiac, adipose, tissue expression, and others). Therefore, it includes basic principles of biological sciences, material chemistry, and relevant engineering subjects. Finally for medical applications after proper clinical verifications of the appropriate films, scaffolds, devices, delivery systems, and other relevant products are needed.

New drugs and innovative devices improve the quality of life for patients with several diseases, which are not necessarily decreased morbidity or mortality in some conditions. Organ replacement is eminently successful but the limitation of available organs made them sparingly used. Tissue engineering and regenerative medicine are proposed as solution by replacing tissue or organ function with constructs that contain specific populations of living cells. Tissue specific stem cells (adult stem cells) become functional cells, which can be used for tissue regeneration (adult stem cell therapy). These cells can replace the defective/damaged cells in different kind of diseases. Therefore, tissue engineering is a new alternate route for the regeneration of damaged/degenerated cells or tissues. This research needs scaffolds (natural and or synthetic), cells (preferably stem cells including induced stem cells), and bioactive molecules (growth hormones). Researchers investigate the possibility of several biomaterials as said using both synthetic and natural substances in the tissue engineering field and regenerative medicines. Different parameters need to be considered before the specific material can be used in clinical investigations.

Due to the advance technology in tissue engineering describing recent findings in this field a few relevant topics are included in this issue. This issue is not a collection of papers based on conferences.

Broad spectrum of tissue engineering-related research work is brought together, for example, potential media supplement for animal cell culture using silk protein sericin, contribution of human umbilical cord blood-derived mesenchymal stem cells towards chondrogenesis, 3D culture of human adult liver cells on hydroxyapatite scaffolds, regenerative peripheral nerve interface for control of a neuroprosthetic limb, and present status in clinical implications of cartilage regeneration in human with adipose tissue-derived stem cells. These areas of research are relevant to the subject of tissue engineering.

It is expected in immediate or near future that there will be development of novel medical devices, artificial organs, cell printing, cell transplantation, and latest combined technologies that will maintain, improve, or restore the functions of diseased organs, new and multifunctional engineered materials, and novel methodological paradigms that challenge advanced thinking in clinical research, applications, and finally valuable assessments.

*Pornanong Aramwit* *Antonella Motta* *Subhas C. Kundu*

